# The association of cardio-metabolic risk factors and history of falling in men with osteosarcopenia: a cross-sectional analysis of Bushehr Elderly Health (BEH) program

**DOI:** 10.1186/s12877-021-02657-1

**Published:** 2022-01-11

**Authors:** Noushin Fahimfar, Shakiba Yousefi, Sima Noorali, Safoora Gharibzadeh, Mahnaz Sanjari, Kazem Khalagi, Ahmad Mehri, Gita Shafiee, Ramin Heshmat, Iraj Nabipour, Azam Amini, Amirhossein Darabi, Gholamreza Heidari, Bagher Larijani, Afshin Ostovar

**Affiliations:** 1 Osteoporosis Research Center, Endocrinology and Metabolism Clinical Sciences Institute, Tehran University of Medical Sciences, Tehran, Iran; 2 Department of Epidemiology and Biostatistics, Pasteur Institute of Iran, Tehran, Iran; 3 Department of Epidemiology, School of Public Health and Safety, Shahid Beheshti University of Medical Science, Tehran, Iran; 4 Chronic Diseases Research Center, Endocrinology and Metabolism Population Sciences Institute, Tehran University of Medical Sciences, Tehran, Iran; 5 The Persian Gulf Marine Biotechnology Research Center, The Persian Gulf Biomedical Sciences Research Institute, Bushehr University of Medical Sciences, Bushehr, Iran; 6 Division of Rheumatology, Department of Internal Medicine, School of Medicine, Bushehr University of Medical Sciences, Bushehr, Iran; 7 The Persian Gulf Tropical Medicine Research Center, The Persian Gulf Biomedical Sciences Research Institute, Bushehr University of Medical Sciences, Bushehr, Iran; 8 Deputy for Education, Ministry of health and medical education, Tehran, Iran; 9 Endocrinology and Metabolism Research Center, Endocrinology and Metabolism Clinical Sciences Institute, Tehran University of Medical Sciences, Tehran, Iran

**Keywords:** Osteosarcopenia, Falling, Elderly, Iran, Cardio-metabolic, Risk factor

## Abstract

**Background:**

Osteosarcopenia, defined as sarcopenia plus osteopenia/osteoporosis, may increase the risk of fractures and affects morbidity and mortality in the older population. Falling is also common in the elderly and increases the risk of fractures and mortality. We examined the association of cardio-metabolic risk factors with a history of falling in osteosarcopenic men.

**Methods:**

We used the baseline data of the Bushehr Elderly Health (BEH) program. Osteosarcopenia was defined as having both sarcopenia (reduced skeletal muscle mass plus low physical performance and/or low muscle strength) and osteopenia/osteoporosis (T-score ≤ − 1.0). Falling was defined as a self-reported history of an unintentional down on the ground during the previous year before the study. We used logistic regression analysis to estimate the adjusted odds ratio (AOR) with a 95% Confidence Interval (CI) to quantify the associations.

**Results:**

All elderly men diagnosed with osteosarcopenia (*n* = 341), with a mean age of 73.3(±7.4) years, were included. Almost 50(14.7%) participants reported falling. Age showed a positive association with falling (AOR: 1.09, 95%CI: 1.04–1.14). An increase of 10 mmHg in systolic blood pressure(SBP), reduces the odds of falling by 26%(AOR:0.74, 95%CI:0.62–0.89), while a positive association was detected for fasting plasma glucose (FPG), as 10 mg/dl increase in the FPG, raises the chance of falling by 14%(AOR = 1.14, 95%CI:1.06,1.23). Hypertriglyceridemia was inversely associated with falling (AOR = 0.33, 95% CI: 0.12, 0.89).

**Conclusions:**

Falling is a major public health problem in rapidly aging countries, especially in individuals with a higher risk of fragility fractures. Older age-raised fasting plasma glucose and low SBP are associated with falling in osteosarcopenic patients.

Considering the higher risk of fracture in osteosarcopenic men, comprehensive strategies are needed to prevent fall-related injuries in this high-risk population.

## Background

Osteopenia/osteoporosis, characterized by low bone mass, together with sarcopenia, characterized by low muscle mass and function, form the new geriatric syndrome, osteosarcopenia [1], that impose a significant burden on the quality of life among the elderly population and a high cost to health systems worldwide [2].

There is a limited number of studies reporting the epidemiology of osteosarcopenia [3]. In Korea, the prevalence of osteosarcopenia in hip fracture patients aged over 60 years was 27.2%, with a 1.8 times higher mortality rate compared to the non-osteosarcopenic individuals [4]. A higher rate was reported from Australia, where it was 40% among a population with a mean age of 80.4 years [5]. The prevalence of osteosarcopenia was recently reported as 34% among elderly aged ≥60 years in Iran [6].

Both bone and muscle mass deterioration share common risk factors in osteosarcopenia [7]. The main risk factors include: 1) mechanical forces, as physical activity has preserved a role for bone and muscle, and inactivity leads to atrophy of both tissues 2) biochemical factors such as hormones include sex hormones, vitamin D, and other factors secreted by muscle and bone, 3) genetic factors, 4) lifestyle behaviors like low vitamin D and protein diet [3, 8].

As a coexistence of two musculoskeletal diseases that are strongly associated with frailty, falls, fractures, hospitalizations, and mortality, osteosarcopenia causes a higher risk of falling and fracture [9, 10]. As noted in previous studies, sarco-osteoporosis had 3.5 times more fracture risk than those without osteoporosis and sarcopenia [11]. Fall is defined as unintentional down on the ground or lower level without any secondary reasons or external forces. It causes significant injuries that are major health problems in the elderly and contributes significantly to morbidity and mortality and imposes high direct and indirect health-related costs [12, 13]. Many risk factors are reported for falls such as age, chronic medical condition, multiple medication use, depression, and cognition impairment. In some studies, the metabolic syndrome had significantly associated with falls [14].

According to the WHO, both genders are at risk for falls, while worldwide, men sustain higher mortality rates and DALYs lost. The higher levels of risk-taking behaviors may be one of the possible explanations of the higher burden detected among men [15].

For many years, osteoporosis has been considered a female gender disease, and male osteoporosis, as a neglected condition, face greater under-diagnosis and under-treatment and deserves more attention [16]. Although osteoporosis is more prevalent in women, some document showed that osteoporosis-related mortality and morbidity rates are higher in men; also with the lower lifetime risk of hip fracture in men, they are twice as likely to die after a hip fracture [17].

Some documents proposed the link between osteoporosis, sarcopenia, and osteosarcopenia with cardio-metabolic risk factors [6, 18–20]. Considering the reasons including 1-the high prevalence of osteosarcopenia in the male elderly population in Iran (33.8%), which puts them at a higher risk of falling (mainly related to the muscle component) and fractures (related mostly to the bone loss), 2-a higher rate of mortality in men due to the falling, and 3-a lower rate of timely diagnosis and treatment of osteoporosis in men, we examined the association between cardio-metabolic risk factors and history of falling in osteosarcopenic men, aged over 60-years living in Bushehr, Iran.

## Materials and methods

### Study population

This is a cross-sectional study that was conducted within the framework of the Bushehr Elderly Health (BEH) program. The BEH program is a prospective, population-based cohort study being conducted since 2013 in Bushehr, southern Iran. The study sample was derived from the elderly urban population. A total of 2426 elderly aged 60 years old and over participated in Stage II of the study which was aimed to investigate musculoskeletal health, cognitive function, and their related risk factors [21]. The cohort study was approved by the Research Ethics Committee of both Bushehr University of Medical Sciences, and Endocrinology and Metabolism Research Institute of Tehran University of Medical Sciences, in accordance with the ethical guidelines outlined in the 1975 Helsinki Declaration.Written informed consents were obtained from all individuals included in the study, after a brief explanation of the study objectives and protocols.

### Data collection

The dual X-ray absorptiometry method was used to measure body composition, muscle mass, and bone mineral density (DXA, Discovery WI, Hologic, Inc., USA). Using a digital dynamometer, muscle strength was measured three times for each hand by handgrip strength. The maximum grip strength was calculated by averaging the highest measurement from both hands. Walking speed (m/s) on a 4.57 m course was considered as an objective measure of physical performance.

Clinical risk factors were collected in the BEH program, using a valid questionnaire with several parts that addressed sociodemographic status, lifestyle behaviors, past medical history, history of falling, drug history, in addition to mental and functional health. The participants underwent a physical examination to collect information on anthropometric measures, blood pressure, gait speed, and handgrip strength. The weight and height were measured by a digital scale and a fixed stadiometer, wearing light clothing and without shoes. Systolic (SBP) and diastolic (DBP) were measured twice on the right arm, after 15 min of rest in the sitting position using a mercury sphygmomanometer. After overnight fasting, venous blood samples were taken, and lipid profile and blood glucose were measured by the enzymatic colorimetric method. The details for the measurements and examinations were described elsewhere [21].

### Definition of terms

The study population consisted of individuals who had both low bone mass and sarcopenia. Low bone mass was defined as T score ≤ − 1 SD below the young adult mean at either femoral neck, lumbar spine, or total hip, considering the Caucasian women aged 20–29 years as the reference group [22]. Sarcopenia was considered a low muscle mass plus low muscle strength or low physical performance [23]. The recommended cutoff value for low skeletal muscle mass was 7 kg/m^2^ in men [24]. Low muscle strength was considered as handgrip strength< 26 kg in men and the cutoff value for physical performance was gait speed< 0.8 m/s [25].

A history of falling was defined as a self-reported unintentional down on the ground or lower level than the previous one [26, 27]. All falling that have been occurred during the previous year before the study were included. Hypertension was considered as SBP ≥ 140 mmHg or DBP ≥ 90 mmHg or taking any antihypertensive medications [28]. The body mass index (BMI) was calculated as weight (kg) divided by squared height (m2). Diabetes was diagnosed with any of these criteria: FPG ≥ 126 mg/dl, HbA1C ≥ 6.5% or taking any anti-diabetic medications [29]. Hypertriglyceridemia was defined as triglyceride level ≥ 150 mg/dl. Hypercholesterolemia and Low HDL-Cholesterol were considered as total cholesterol ≥200 mg/dl and HDL-C < 40 mg/dl, respectively [30]. Lifestyle was categorized to four groups based on the physical activity level as sedentary (1–1.39), low active (1.4–1.59), active (1.6–1.89), and very active (1.9–2.5) [31]. We defined low physical activity, considering the sedentary and low active. High physical activity includes active and very active individuals. Smoking was defined as smoking water pipes or cigarettes at the study time.

### Statistical analysis

Summary statistics were explained as the frequency (percentage) for categorical variables and mean (± standard deviation) for normally distributed continuous variables. Variables with non-normal distribution diagnosed by the Shapiro-Wilk normality test were presented as the median and interquartile range (IQR). We compared the individuals with and without a history of falling for the baseline characteristics using Pearson’s Chi-square test for the categorical variables. Independent sample t-test and Mann-Whitney-u test were used for normally and non-normally distributed variables, respectively. Among potential confounding variables including age, education, FPG, SBP, lipid profiles, current smoking, BMI, waist circumference, and physical activity, the best subset included age, SBP, FPG, and hypertriglyceridemia, selected by the Akaike Information Criterion (AIC). We used multivariable fractional polynomials (MFP) to check the non-linearity of predictor – outcome associations [32]. We used the logistic regression model to determine the association of the potential cardio-metabolic variables and history of falling (as dependent variable) in individuals with osteosarcopenia. The interaction terms between age and other risk factors were assessed. The receiver operating characteristic (ROC) curve was used to show the goodness of fit of the final model. All the *p*-values < 0.05 were considered as statistically significant. Stata statistical package (*Stata Statistical Software: Release 14*. College Station, TX: StataCorp LP) was used to perform the statistical analyses.

## Results

This study was performed using the data of all osteosarcopenic men (*n* = 341) in BEH program. Among them, 50 (14.7%) individuals reported a positive history of falling. Table 1 shows the baseline characteristics of the participants by a history of falling. On average, participants with a history of falling were 3 years older than the others with no history of falling (75.9 vs 72.9 years, *P* = 0.007). Systolic Blood Pressure was also significantly different in individuals with and without a history of falling. Hypertriglyceridemia showed a higher prevalence in participants without a history of falling; however, the difference was not statistically significant (24.4% vs 12.0%, *P* = 0.053). There was no difference in the mean values of BMI in patients with and without a history of falling (24.0 vs 23.9, *P* = 0.848).

**Table 1:** Baseline characteristics of the participants by history of falling; Bushehr Elderly Health BEH) program

	Fall + ***N*** = 50	Fall – ***N*** = 291	***P***-value
**Continuous Variable**	**Mean (SD)**	**Mean (SD)**	–
Age, year	75.9 (7.9)	72.9 (7.3)	0.007
Education^a^, Year	6 (1–10)	6 (0–10)	0.849
Systolic Blood Pressure, mmHg	132.1 (16.0)	141.1 (21.3)	0.005
Body Mass Index, kg/m^2	24.0 (3.9)	23.9 (3.3)	0.848
Waist Circumference, cm	92.3 (12.2)	91.9 (10.3)	0.799
Fasting Plasma Glucose^a^, mg/dl	91.5 (81.3–131.8)	88 (82–100)	0.423
**Categorical variables**	**N (%)**	N (%)	–
Current smoking	9 (18.0)	72 (24.7)	0.301
Diabetes	19 (38.0)	80 (27.6)	0.134
Hypertension	34 (68.0)	204 (70.1)	0.765
Hypertriglyceridemia	6 (12.0)	71 (24.4)	0.053
Hypercholesterolemia	12 (24.0)	70 (24.1)	0.993
Low HDL-Cholesterol	19 (38.0)	107 (36.8)	0.868
Physical activity	5 (10.0)	41 (14.1)	0.611

- Diabetes was defined as FPG ≥ 126 mg/dl, or HbA1C ≥ 6.5 or taking anti-diabetic medication

- Hypercholesterolemia was defined as total cholesterol ≥200 mg/dl

- Low HDL-Cholesterol was defined as HDL-C < 40 mg/dl

- Hypertriglyceridemia was defined as triglyceride ≥150 mg/dl

^a^ Due to not normal distribution, median (25th–75th percentile) was reported and *p*-value was estimated using non-parametric test

Table 2 shows the determinants of falling in men with osteosarcomopenia, adjusted for other variables. Falling was significantly associated with age, SBP, FPG level in patients under treatment, and hypertriglyceridemia. As shown, there was a positive association between the presence of falling and age (OR = 1.09, CI 95% = 1.04–1.14). History of falling was inversely associated with SBP, (OR = 0.74, CI 95% = 0.62–0.89). The FPG level was positively related to the history of falling, as a 10 mg/dl increase in the level of FPG increased the chance of falling by 14% (OR = 1.14, 95%CI = 1.06–1.23). Hypertriglyceridemia was inversely associated with falling (OR = 0.33, CI 95% = 0.12 to 0.89).

**Table 2:** Determinants of falling in men with osteosarcopenia; Bushehr Elderly Health (BEH) program

	Odds Ratio	95% CI^**a**^	***P***-value
Age, year	1.09	1.04–1.14	< 0.001
Systolic Blood Pressure, 10 mmHg	0.74	0.62–0.89	0.001
Fasting Plasma Glucose, 10 mg/dl	1.14	1.06–1.23	< 0.001
Hypertriglyceridemia	0.33	0.12–0.89	0.028

^a^*CI* Confidence Interval

**Fig. 1:**
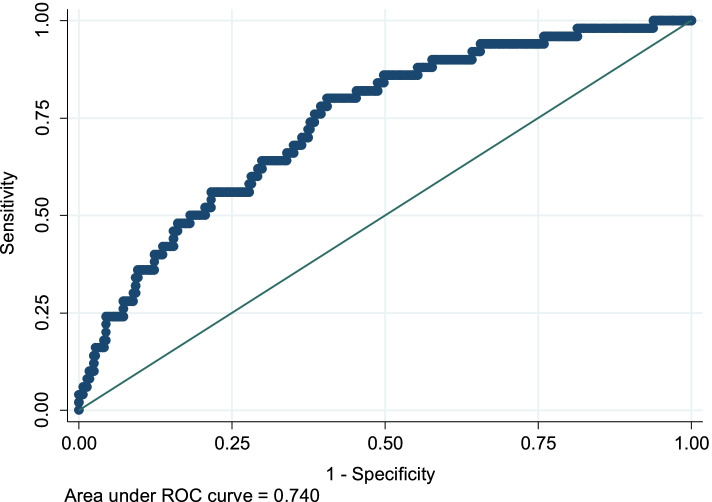
The receiver operating characteristic (ROC) curve showing the discrimination power of variables correlates to the falling in men with osteosarcopenia

The ROC curve replicating the discrimination power of the multivariable logistic regression model was presented in Fig. 1. The area under the ROC curve showed a discriminative ability of 74.04% for the included variables.

## Discussion

The present study examined the association of cardio-metabolic risk factors and falling in a population of Iranian osteosarcopenic men. The result of the study showed that age and FPG had a positive association with falls, whereas we observed the inverse association of SBP and TG. The other risk factors were not significantly associated with an increased risk of falling in the osteosarcopenic people.

Considering the high rates of mortality after both falling and fractures, this study aimed to investigate the associates of falling in osteosarcopenic men. Not only a lower rate of correct and timely diagnosis of osteoporosis were reported in men, fewer men take adequate treatment compared to women.

In our study, the presence of falls was strongly correlated with age in osteosarcopenic men. Many documents suggest aging are accompanied by osteoporosis, sarcopenia, and both of them are common diseases that predominantly affect the elderly and considered as risk factors for dysmobility, frailty, falls, fractures, and mortality in older people [33–35].

It has been shown that uncontrolled blood pressure increases the risk of immediate standing balance impairment. Some studies showed the effect of a drop in blood pressure on increasing the rate of falls [36–39]. Our study revealed that SBP (including treated or untreated status) is inversely associated with falling. In line with our project, Klein et al. showed that an increase of SBP by ten mmHg reduced the risk of falling by 9% in women [36]. Orthostatic hypotension (OH), as a risk factor for falls, is a common clinical phenomenon in the elderly population and is associated with blood pressure treatment [40]; so, the management of hypertension in the elderly population requires a careful balancing of the benefits and risks of therapy [36–39, 41]. Considering the high prevalence of both osteosarcopenia (34%) [6] and hypertension (42%) in the elderly population [42], comprehensive care for hypertension is needed.

In our study, there was also a remarkable correlation between the FPG level and fall in men with osteosarcopenia. Type 2 diabetes (T2D) causes more falls due to different factors such as peripheral and autonomic neuropathy, retinopathy, microvascular complications, and other comorbidities like obesity [14] and muscle dysfunction [43] with 30% lower leg muscle strength in diabetics compared to the non-diabetic group [44]. Insulin resistance and glycation end-products (AGEs) accumulation can cause losses in muscle mass and strength, resulting in the sarcopenia and plays an essential role in the interaction between sarcopenia and T2D [45–47]. Another study showed that hypoglycemia increases the risk of falls in diabetic patients due to the decrease in attention, slowing psychomotor speed, and impairment of vascular autonomic nerves [48]. Although many studies showed that diabetes and its complications play essential roles in falling, studies about the direct association between blood sugar and fall were insufficient, especially in osteosarcopenia. Cesare Berra and colleagues also showed that both hypoglycemia (BS < 70 mg/dl) and hyperglycemia (BS > 200 mg /dl) were significantly associated with increased risk of falls among hospitalized patients [49]. In our study, there was also a remarkable correlation between the FPG level and fall in men with osteosarcopenia. We found that increasing FPG can significantly associate with the history of falling in patient with osteosarcopenia and should be carefully controlled.

In our study, there was no association between falls and BMI in osteosarcopenic men. The result of a meta-analysis revealed that the risk of falling increases in the obese older population aged over 60 years by 16%, compared to the non-obese ones, and that was due to poor lower limb muscle quality, increase foot load, and impaired postural control [15]; however, another study showed that both sarcopenia and obesity have an independent association with fall [50]. Researchers explained that these different results might be due to using various definitions of obesity, such as BMI, waist circumference, or assessment of body fat mass. In the current study, the mean values of BMI were the same in participants with and without a history of falling, resulting in less heterogeneity in the study population, which may explain the lack of association.

We found that hypertriglyceridemia can reduce the chances of falling by almost 60%. To our knowledge, there is no study to show the association between lipid profile and falls in osteosarcopenic patients, while adjusted by other cardiovascular risk factors, TG > 150 mg/dl showed an inverse association with pre-frailty (OR: 0.61) and frailty (OR: 0.49) among hospitalized older adults in Brazil, although these associations were not statistically significant [51]. Low levels of triglycerides might be potential warning signals of problems in nutritional status or rapidly deteriorating health, while more data are needed to clarify the association between triglycerides and falling in the older population.

This research, with a sample from a population-based study, supplied information on factors associated with falling in osteosarcopenic patients. Some limitations should be acknowledged; osteosarcopenia is a newly described syndrome, and comprises of two diseases; so, the results identified can be influenced by the direction and also the power of the association of variables with each of the disease components. Since studies that directly target the osteosarcopenia are scarce, comprehensive comparison with other studies is not entirely possible. 

Moreover, our research was a cross-sectional studآy, and the result did not interpret any causal relations. Further prospective studies are required to clearly explain the leading risk factors of falling in osteosarcopenic patients. Considering the cross-sectional design of the study, the generalizability of the results should be further assessed in other studies with a different population in a larger sample size.

## Conclusion

Age and FPG levels showed a positive association with history of falling in osteosarcopenic men, while an inverse association was detected for SBP and TG. Considering both the role of falling in inducing osteoporotic fractures, and the possible increased risk of fractures in osteosarcopenic patients, and also the higher rate of mortality after falling and fractures in elderly men, comprehensive care is needed to control the cardiometabolic risk factors, especially blood pressure in men with osteosarcopenia.

## Data Availability

The datasets used and/or analysed during the current study are available from the corresponding author on reasonable request.

## Abbreviations

BEH: Bushehr Elderly Health
AOR: Adjusted Odds Ratio
DXA: Dual X-ray absorptiometry
CI: Confidence Interval
SBP: Systolic Blood Pressure
FPG: Fasting Plasma Glucose
DBP: Diastolic Blood Pressure
BMI: Body Mass Index
IQR: Interquartile Range
AIC: Akaike Information Criterion
MFP: Multivariable Fractional Polynomials
ROC: Receiver Operating Characteristic Curve
T2D: Type 2 diabetes
TG: Triglyceride
